# Analysis of aspartate aminotransferase-to-platelet ratio index and liver fibrosis in biliary atresia

**DOI:** 10.4102/jcmsa.v4i1.447

**Published:** 2026-08-17

**Authors:** Elizabeth Brits, Stephen Brown, Lezelle Botes, Michael Pienaar

**Affiliations:** 1 Department of Surgery, Faculty of Health Sciences, University of the Free State, Bloemfontein, South Africa; 2 Department of Paediatrics and Child Health, Faculty of Health Sciences, University of the Free State, Bloemfontein, South Africa; 3 Department of Health Sciences, Faculty of Health and Environmental Sciences, Central University of Technology, Bloemfontein, South Africa

**Keywords:** biliary atresia, liver injury, aspartate aminotransferase-to-platelet ratio index, METAVIR fibrosis score, biomarker, outcome, prognosis

## Abstract

**Background:**

The aspartate aminotransferase-to-platelet ratio index (APRi) has been proposed as a non-invasive biomarker of liver injury in biliary atresia (BA). However, conflicting evidence and varying cutoff values have left its association with histologically confirmed liver damage uncertain. This study assessed the relationship between APRi and liver injury severity (Meta-Analysis of Histological Data in Viral Hepatitis [METAVIR] F0–F4) in BA and evaluated its predictive value.

**Methods:**

A retrospective analytical review of electronic records was conducted for all BA patients treated at a South African academic hospital between 01 January 2009 and 31 December 2019.

**Results:**

Sixty-seven patients were included, 74.6% of whom were female. The most common subtypes were cytomegalovirus (CMV) immunoglobulin M-positive (IgM+) BA (34.3%) and isolated BA (31.3%); about one-third could not be classified because CMV serology was unavailable. Liver biopsy METAVIR scores were F1 (8.3%), F2 (50.0%), F3 (18.3%) and F4 (23.3%). In a sub-analysis (*n* = 39), APRi modestly differentiated ≤ F2 from ≥ F3 fibrosis at a cutoff of 2.69 (area under the receiver operating characteristic curve 0.61). Decision curve analysis suggested modest clinical utility despite a non-significant logistic regression model (odds ratio 1.25; 95% confidence interval 0.94–1.92; *p* = 0.2). Of the 55 patients with known outcomes, 94.5% (*n* = 52) had confirmed mortality or were referred for palliative care.

**Conclusion:**

Aminotransferase-to-platelet ratio index should not replace established clinical decision-making but may provide useful adjunctive information alongside clinical, biochemical and histological assessment. These findings provide contextual evidence from a South African resource-limited setting and support prospective multicentre validation before routine clinical implementation.

**Contribution:**

The study contributes evidence supporting continued evaluation of APRi as an adjunctive non-invasive biomarker in BA in the South African setting.

## Introduction

Biliary atresia (BA) is a rare condition in neonates where the bile ducts are blocked, leading to cholestasis and progressive liver damage.^1,2^ When untreated, it can be fatal within 3 years due to liver failure.^3^ The two main treatment options are Kasai hepatoportoenterostomy (KPE)^4^ and liver transplantation (LT).^5^ While KPE may prolong survival with the native liver, its long-term success varies widely.^6^ Many patients still require LT before adulthood due to progressing liver fibrosis.^7^ It remains unclear why bile flow is restored in some patients after KPE, while others rapidly progress to end-stage liver disease.^2^ One potential explanation might be the degree of initial liver injury at the time of performing KPE.^8^ An LT is crucial for patients with established significant liver injury.^9^

Performing KPE before 70 days of age is recommended for optimal outcomes.^10^ Successful procedures on infants over 100 days of age are rare.^11^ If the patient is older than 120 days or has advanced fibrosis (Meta-Analysis of Histological Data in Viral Hepatitis [METAVIR] ≥ F3/unfavourable histology), a primary LT will be suggested instead of a KPE.^12^ Due to delayed referrals and ensuing liver damage, the question arises whether to proceed with KPE or directly refer for LT evaluation without KPE.^13^ Determining the extent beforehand would be advantageous, avoiding unnecessary major procedures and saving valuable time and resources.^13,14^

A liver biopsy is the gold standard for evaluating liver injury.^15^ Nevertheless, it has practical drawbacks, such as its invasive nature, need for sedation, regional sampling error, inter- and intra-observer variation of interpretation and potential complications.^16,17^ Non-invasive diagnostic tools are needed for management decisions and follow-up assessments,^16,18^ including imaging techniques, serum biomarkers, indices and combination panels.^17,19,20,21,22^

Limited studies correlate liver stiffness with liver injury severity and KPE prognosis in BA using imaging techniques. The relationship between the imaging-detected degree of liver injury and the postoperative outcome of KPE remains undecided.^22^ These techniques are not ideal diagnostic and prognostic tools due to their limitations, including cost, need for sedation in paediatric patients, longer length of time for magnetic resonance (MR) elastography and possible decreased accuracy from other aetiologies such as iron overload, vascular congestion, steatosis, cholestasis and portal hypertension.^23^

Non-invasive indices and scores have been proposed to predict liver injury in BA. These include hepatic dysfunction scores such as the Child–Turcotte–Pugh score,^24^ paediatric model of end-stage liver disease score^25^ and the Mayo Clinic updated natural history model score.^26^ These scores are not sufficiently sensitive to predict hepatic fibrosis except in patients with cirrhosis and portal hypertension.^24,25^ However, another tool developed to address difficulties in managing adult patients with hepatitis C-related chronic liver disease is the aspartate aminotransferase-to-platelet ratio index (APRi).^27^

Although APRi has been evaluated internationally as a marker of fibrosis severity^11,20,21,28,29^ and prognosis in BA, evidence from African and resource-constrained healthcare settings remains limited. This study therefore examined the association between APRi and histological liver fibrosis, assessed using the METAVIR score,^14,15^ in a South African BA cohort characterised by delayed presentation, a high burden of CMV IgM-positive disease, human immunodeficiency virus (HIV) exposure and limited access to LT. Within this context, the study also explored whether APRi could provide adjunctive information to support clinical decision-making regarding the severity of liver injury, while recognising that APRi is not intended to replace established clinical, histological or surgical assessment.^14^

## Research methods and design

### Study context

The Universitas Academic Hospital Complex (UAHC) provides paediatric surgical care to central South Africa (Free State, Northern Cape, parts of Eastern Cape) and Lesotho, with extensive cross-provincial and cross-border referrals. In South Africa, BA management is decentralised. The UAHC treats our patient population with early-diagnosed BA (favourable liver histology and ≤ 120 days) using KPE.^12^ Standard recommendations advise performing a KPE within 90 days of life.^10,11^ However, our unit considers patients up to 120 days, including those over 100 days who may still benefit.^12^ Patients with failed KPE or liver cirrhosis are referred to the LT team at Wits Donald Gordon Medical Centre, University of the Witwatersrand, Johannesburg, South Africa.

### Study sample and measurement

This study was a retrospective analytical record review of all 67 patients diagnosed with and treated for BA at the UAHC from 01 January 2009 until 31 December 2019. Information was collected from patients’ electronic records and managed using REDCap electronic data capture tools (REDCap Consortium; Nashville, TN, USA).

The following information was captured:

- Demographic data (sex, age at diagnosis).
- Type of BA: Cystic BA (CBA), isolated BA (IBA), cytomegalovirus-associated BA (CMV IgM + BA), BA splenic malformation syndrome (BASM). A positive CMV IgM was used to confirm CMV IgM + BA in this study due to the difficulty in establishing a histological diagnosis, and the fact that CMV viral load tests were unavailable at our institution during the study period.
- Blood results peri-liver biopsy or at the time of presentation at UAHC if no biopsy was done, including aminotransferases and platelet count and calculation of the APRi value.^26,27^
- Degree of histological liver injury at the time of diagnosis or KPE according to the METAVIR fibrosis score (F0 = no fibrosis, F1 = portal fibrosis, F2 = peri-portal fibrosis, F3 = septal fibrosis, F4 = cirrhosis).^15^
- Outcome, as determined at the end of the study period. Patient mortality was defined as confirmed demise documented in clinical records or as an official down-referral for palliative care, ensuring that outcomes were verified rather than assumed.

### Data analysis

The Department of Biostatistics, Faculty of Health Sciences, University of the Free State (UFS), analysed data using SAS software version 9.4. (SAS Institute, Inc.; Cary, NC, United States). Descriptive statistics, namely medians and percentiles, were calculated for numerical variables. Frequencies and percentages were calculated for categorical data. The cutoff APRi values for the association of different degrees of liver fibrosis were assessed using the receiver operator characteristic (ROC) curve compared to the pathological METAVIR fibrosis score of the liver.

The APRi was calculated as follows^27^:

APRi = aspartate aminotransferase (AST) (IU/L)/AST upper normal limit (IU/L) (= 40)/platelet count (per 10^9^/L) × 100

Decision curve analysis (DCA) was conducted to evaluate the clinical utility of the APRi value in predicting advanced liver disease in infants with BA aged ≤ 120 days at diagnosis, aiding in choosing between evaluation for a primary LT or KPE.^30^ The analysis incorporated a logistic regression model that assigned a probability score for the extent of liver damage based on the APRi value at diagnosis.

### Ethical considerations

Ethical clearance to conduct this study was obtained from the University of the Free State Health Sciences Research Ethics Committee (No. UFS-HSD2022/1889/2504). Permission to conduct the study was obtained from the Free State Department of Health. Due to the retrospective nature of the study and using only archived patient records to collect data, obtaining informed consent from patients’ parents and/or guardians was not required.

## Results

### Patient demographics

This study included 67 patients with a median age of 107 days (range 39–358 days). Most patients were female (*n* = 50; 74.6%). Eighteen (26.9%) patients were HIV-exposed, and one (1.5%) tested positive for HIV. There was no association between the median age at BA diagnosis and the APRi value (median 1.86, range 0.48–7.31) (*r* = 0.336). Similarly, in patients younger than 120 days, no association between age and their APRi values was observed (median 1.65, interquartile range [IQR] 0.825–2.44) (*p* = 0.05). The median APRi score in the HIV-exposed patients (median 1.650, IQR 0.860–2.48) was not significantly higher than in the unexposed patients (median 1.940, IQR 1.085–2.790).

### Surgical management and aminotransferase-to-platelet ratio index

Thirty-two (47.8%) patients underwent KPE at a median age of 76 days (range 39–148). The 35 (52.2%) patients who did not undergo the procedure had a median age of 138 days (range 85–358). This difference was statistically significant (*p* < 0.0001). The patients who underwent KPE had a significant correlation between the APRi value (median 1.2, range 0.5–15) and age at KPE (*p* = 0.04035), a lower APRi value than infants who did not undergo KPE. In contrast, no correlation was found between the APRi value (median 2.5, range 0.66–27.3) and the age at diagnosis when KPE was not performed (*p* = 0.12208), in which case the APRi value was higher.

### Type of biliary atresia and histological findings

The findings regarding the type of BA and histological evaluation (METAVIR scores) are summarised in Table 1. A similar number of patients were diagnosed with IBA and CMV IgM + BA (*n* = 21, 31.3% and *n* = 23, 34.3%, respectively). In 21 (31.3%) patients, the lack of CMV serology data resulted in not assigning a type of BA and classification was indicated as unknown. Consequently, a correlation between APRi and the type of BA was not determined. Fifteen (22.4%) patients had other congenital abnormalities, including cardiac lesions (*n* = 3; 4.5%), polysplenia (*n* = 2; 3.0%), malrotation (*n* = 2; 3.0%) and situs inversus (*n* = 1; 1.5%).

**TABLE 1:** Distribution of patients regarding type of biliary atresia and histological evaluation.

Variable	*n*	%
Type of biliary atresia (*n* = 67)		
Isolated BA (IBA)	21	31.3
BA splenic malformation syndrome (BASM)	2	3.0
Cytomegalovirus IgM + BA (CMV IgM + BA)	23	34.3
Cystic BA (CBA)	0	0.0
No type assigned; classified as unknown	21	31.3
Histological evaluation (METAVIR score) (*n* = 60)		
F0: No fibrosis	0	0.0
F1: Portal fibrosis	5	8.3
F2: Peri-portal fibrosis	30	50.0
F3: Septal fibrosis	11	18.3
F4: Cirrhosis	14	23.3

CMV, cytomegalovirus; IgM, immunoglobulin M-positive; BA, biliary atresia.

Sixty (89.6%) patients underwent a liver biopsy with histological evaluation at KPE or at the time of diagnosing BA. The METAVIR scores of these patients are shown in Table 1. Thirty-five (58.3%) patients had early fibrosis (≤ F2), while advanced fibrosis or cirrhosis (≥ F3) was identified in the remaining 25 (41.7%) patients.

### Laboratory values

The median AST level was 272.0 U/L, and the median platelet level was 433.0 × 10^9^/L. The median APRi ratio was calculated at 1.86 (range 0.48–27.31). Table 2 presents the median, minimum and maximum results of pre-operative and/or diagnostic tests.^12^

**TABLE 2:** Results from pre-operative and/or diagnostic tests.

Pre-operative blood results (*n* = 67)	Median	Range
Platelet count (×10^9^/L)	433.0	27.0–907.0
Aspartate aminotransferase (AST) (U/L)	272.0	69.0–892.0
Alanine aminotransferase (ALT) (U/L)	143.0	25.0–557.0
Alkaline phosphatase (ALP) (U/L)	811.0	358.0–2456.0
Gamma-glutamyl transferase (GGT) (U/L)	823.0	55.0–3338.0
Bilirubin		
Total (µmol/L)	195.0	93.0–362.0
Conjugated (µmol/L)	162.0	71.0–324.0
Albumin (g/L)	29.0	13.0–42.0
International normalised ratio (INR)	1.20	0.93–10.0

*Source:* Brits E, Le Grange SM. Biliary atresia: The profile, management and outcome of patients treated at a tertiary hospital in central South Africa. S Afr Med J. 2023;113(11):57–62. https://doi.org/10.7196/SAMJ.2023.v113i11.845

### Aminotransferase-to-platelet ratio index values and METAVIR scores

As shown in Table 3, the APRi values associated with the different METAVIR scores (F0–F4) were calculated for the total study population. A statistically significant association between APRi and METAVIR score was found (*p* = 0.0336). A sub-analysis of 39 patients younger than 120 days of age, who had METAVIR scores based on liver biopsy findings, was performed. Of these patients, 28 (71.8%) had favourable histology (METAVIR ≤ F2) with a median APRi value of 1.42 (IQR 0.82–2.42), while the remaining 11 (28.2%) had unfavourable histology (METAVIR ≥ F3) with a median APRi value of 1.66 (IQR 0.79–3.71), which was not statistically significant (*p* = 0.3332).

**TABLE 3:** Results of aminotransferase-to-platelet ratio index value at different METAVIR scores.

METAVIR score	Total patients with liver histology† (*n* = 60)				Patients ≤ 120 days with liver histology‡ (*n* = 39)			
	*n*	%	Median APRi	IQR	*n*	%	Median APRi	IQR
F0	0	-	-	-	0	-	-	-
F1	5	8.3	0.78	0.64–1.43	5	12.8	0.78	0.64–1.43
F2	30	50.0	1.78	0.89–2.48	23	59.0	1.75	0.89–2.44
F3	11	18.3	1.66	1.34–2.51	8	20.5	1.65	1.185–2.49
F4	14	23.3	2.69	1.92–3.71	3	7.7	3.71	0.79–4.39

APRi, aminotransferase-to-platelet ratio index; IQR, interquartile range.

† , *p* = 0.0336; ‡, *p* = 0.2979.

An APRi value of 2.69 was identified as the cutoff value to distinguish the two histological groups. The area under the receiver operating characteristic curve (area under curve [AUC]) for APRi was 0.61, with a 95% confidence interval (CI) of 0.55–0.67. The positive predictive value (PPV) was 77.4%, the negative predictive value (NPV) was 50.0%, sensitivity was 85.7% and specificity was 36.4% for unfavourable histology. The Youden Index, assessing the balance between sensitivity and specificity, was 22.1%.

### Decision curve analysis of the aminotransferase-to-platelet ratio index value predictive model for liver damage

**FIGURE 1:**
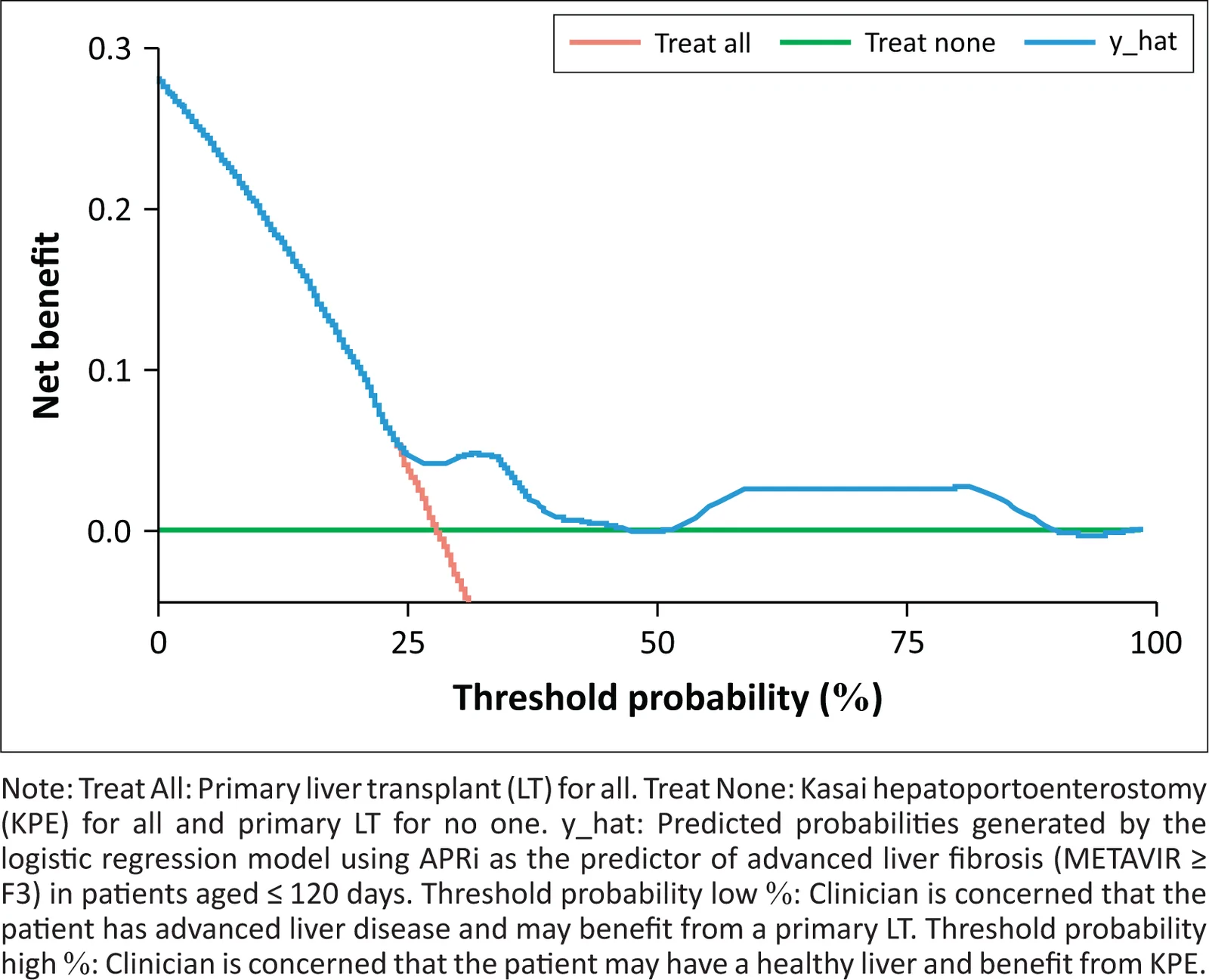
Decision curve analysis of the aminotransferase-to-platelet ratio index value predictive model for liver damage.

Figure 1 presents the decision curve analysis of the logistic regression model using APRi to predict advanced liver fibrosis. The model’s predicted probabilities (y_hat) were compared with the default strategies of performing primary LT for all patients (‘Treat All’) or KPE for all patients (‘Treat None’). The odds ratio (OR) for the APRi value was 1.25 (95% CI 0.94–1.92, *p* = 0.2).

### Outcome

**FIGURE 2:**
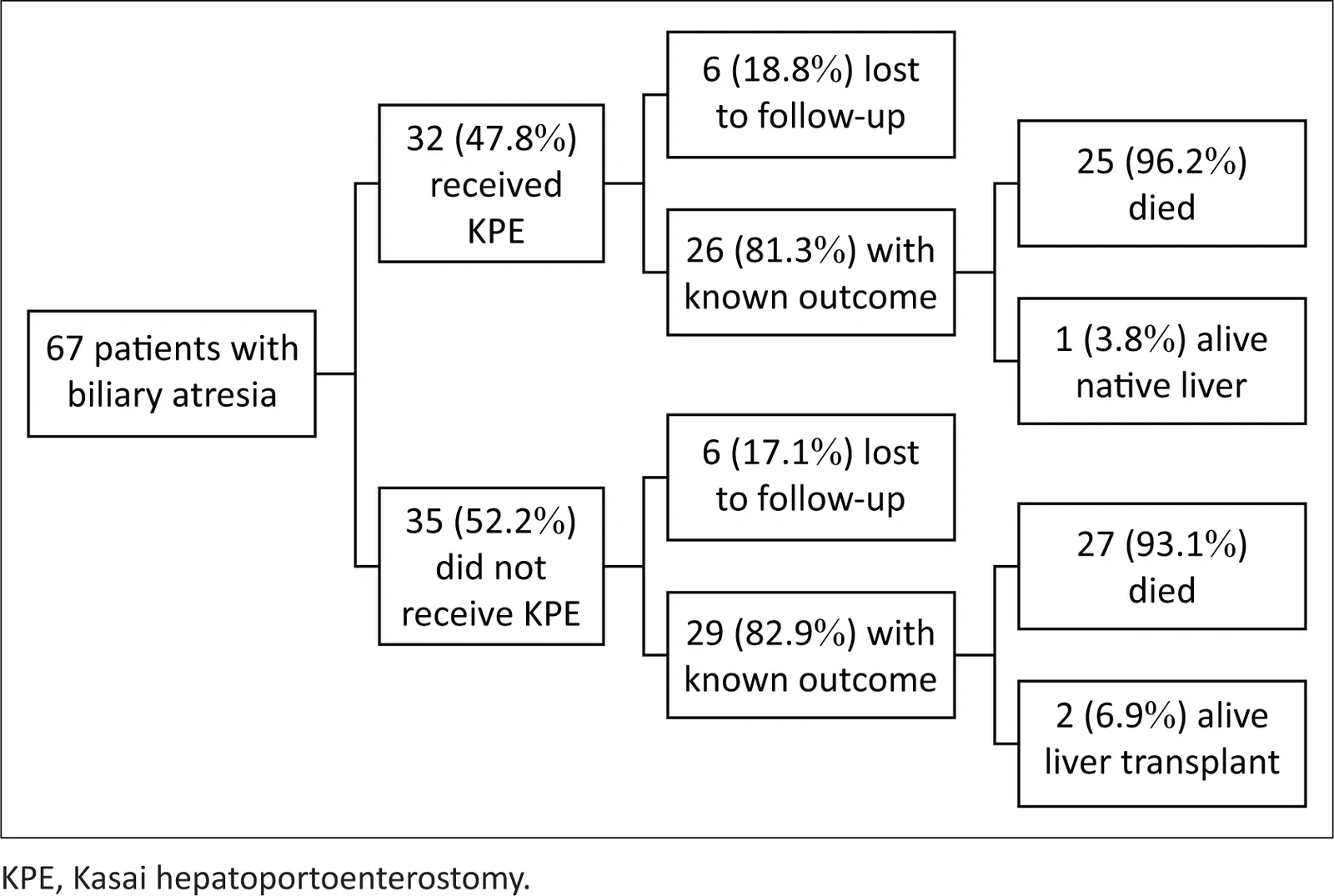
Diagrammatic presentation of the outcomes of patients with biliary atresia.

The diagram presented in Figure 2 illustrates the outcomes of patients with BA in this study. Twelve patients (17.9%) were lost to follow-up, and their outcomes were unknown. Of the 55 patients with a known outcome, 52 (94.5%) died after receiving a KPE or palliative treatment only, and three (5.5%) were alive at the end of the study period. Of the three survivors, one had a KPE (1-year native liver survival [NLS] rate), and two had LTs without a prior KPE (5-year survival 100%). No predictive value or correlation with APRi was calculated due to the poor outcome.

## Discussion

This study examined the relationship between APRi and histological liver fibrosis in a South African cohort of infants with BA managed in a resource-limited healthcare setting. Although APRi has previously been investigated in Europe and Asia, evidence from African populations remains limited. Our findings add contextual evidence from a cohort characterised by delayed presentation, a high prevalence of CMV IgM-positive disease, HIV exposure and limited access to LT. Consistent with previous studies,^21,31,32,33^ aminotransferase-to-platelet ratio index showed only modest discrimination for advanced fibrosis, supporting its use as a potential adjunctive biomarker rather than a standalone test for clinical decision-making. The study therefore provides regional validation and highlights the challenges of applying non-invasive fibrosis markers in settings where access to specialist investigations and transplantation remains limited.

### Aminotransferase-to-platelet ratio index value predictive model

The study aimed to determine a cutoff value for APRi (2.69) and assess its diagnostic accuracy in detecting unfavourable histology in a cohort of patients with BA aged 120 days or younger. Our findings showed that the accuracy of APRi in diagnosing unfavourable histology was limited, with an AUC of 0.61 and an optimal threshold Youden Index of 22.1%. Studies have indicated strong positive correlations between APRi and patient age,^19,20^ bilirubin levels,^19^ spleen size^19^ and fibrosis severity,^21,34,35^ However, cutoff values vary across different studies.^19,21,34^ Further prospective validation is required before APRi can be considered a reliable surrogate marker for assessing the severity of liver fibrosis in BA.

The decision curve analysis (Figure 1) showed variation in net benefit across threshold probabilities when comparing decisions between primary LT and KPE. The APRi-based model suggested a modest potential net clinical benefit across most threshold probabilities compared with the default ‘treat-all’ and ‘treat-none’ strategies. This indicates that APRi may provide additional information to support clinical decision-making when interpreted alongside established clinical assessment.

Between threshold probabilities of 46% and 54%, the model showed no net benefit, consistent with the underlying logistic regression model (OR 1.25; 95% CI 0.94–1.92; *p* = 0.2). This finding reflects the model’s limited discriminatory ability within this threshold range and reinforces the need for cautious interpretation.

Overall, the decision curve analysis suggests that the APRi-based model may have modest clinical utility. However, the observed benefit was small, discriminatory performance was limited (AUC 0.61), and the logistic regression model was not statistically significant. These findings should therefore be interpreted as exploratory and hypothesis-generating, rather than as definitive evidence supporting routine clinical implementation.

### Aminotransferase-to-platelet ratio index value and METAVIR fibrosis score

We found an association between APRi and the METAVIR score for the total study population. However, no association was found between the APRi and the METAVIR score at the time of diagnosis or KPE in patients aged ≤ 120 days. This observation might be attributed to the smaller sample size of the younger patients, adding to conflicting published information and limited data regarding the relationship between APRi and METAVIR scores at diagnosis or KPE.^19,20,21,28,34,35^

Some smaller studies could not establish a significant association.^20,28^ In comparison, larger studies reported a significant association with different cutoff values for APRi at the time of BA diagnosis and degree of liver fibrosis.^19,21,34,35^ More extensive prospective multicentre studies or meta-analyses are recommended to address contradictory findings in the literature and determine a universal APRi cutoff value for advanced fibrosis or cirrhosis (≥ F3), potentially reducing unnecessary procedures in older patients diagnosed with BA.

### Age

Our results demonstrated no direct link between APRi and the patient’s age at BA diagnosis for the total study population (median age 107 days). However, a significant correlation was found between APRi and age at KPE (median age 76 days). This correlation was consistent with research conducted in the United Kingdom^19^ and Finland.^20^ Therefore, APRi seems more accurate in BA patients at a younger age.

### Human immunodeficiency virus and cytomegalovirus

Further exploration is necessary to understand the reasons behind our study’s elevated APRi value of 2.69. One potential factor is the high rate of HIV exposure, maternal antiretroviral therapy and neonatal antiretroviral prophylaxis in our study population, which might have caused liver function abnormalities.^36,37^ The use of these drugs was not formally documented in this study. Another potential factor is our study population’s high CMV IgM + BA cases, which might have contributed to this high APRi value.^38,39^ While our study found high APRi values, it is unclear if it were directly related to HIV exposure, antiretroviral prophylaxis or CMV IgM positivity. These factors may be confounding variables affecting liver function. Future studies should also consider other factors that may independently influence APRi values, including intercurrent hepatic inflammation, thrombocytopenia from causes unrelated to liver fibrosis and other conditions affecting serum aminotransferase levels or platelet counts.

### Outcome

More than half of our patients presented late (after the standard period indicated for performing a KPE), with subsequent poor outcomes, as evident in our results and reported previously.^12^ This emphasises the importance of promoting awareness and timely referrals. Considering our poor patient outcomes, we were motivated to do this study to develop this non-invasive APRi prediction model. We cannot expose our patient population to failing major surgery (KPEs) simply because we have limited access to LT and new diagnostic modalities (e.g. elastography), knowing our poor outcomes. Although these findings support the continued evaluation of APRi in resource-limited healthcare settings, they should be interpreted within the context of the study’s methodological and clinical limitations.

### Strengths and limitations

This study has several strengths. It represents one of the few evaluations of APRi in a South African BA population and provides contextual evidence from a resource-limited healthcare setting characterised by delayed presentation, a high prevalence of CMV IgM-positive disease, HIV exposure and limited access to LT. The inclusion of decision curve analysis extends the evaluation beyond conventional measures of diagnostic accuracy by examining the potential clinical utility of an APRi-based prediction model.

Several limitations should also be acknowledged. The retrospective single-centre design and the lack of liver histology for all patients may have introduced selection bias and may limit the generalisability of the findings. Although this study included one of the larger South African BA cohorts reported to date, the sample size remained relatively small for developing and evaluating a predictive model.

Aminotransferase-to-platelet ratio index was evaluated as a single biomarker, and comparative analyses with other routinely available biochemical markers or formal multivariable prediction models were beyond the scope of the present study. Similarly, subgroup analyses according to CMV status and HIV exposure were limited by missing CMV serology and the relatively small sample size.

An additional limitation concerns the interpretation of the decision curve analysis. This type of analysis is most informative when the competing management strategies are both realistically available and adequately represented within the study population. In our setting, access to LT was extremely limited, with only a small number of patients ultimately undergoing transplantation, and overall poor clinical outcomes. Consequently, although the decision curve analysis provides an estimate of the potential clinical utility of the APRi-based model, these findings should be interpreted cautiously and may not be directly generalisable to centres with well-established LT programmes.

Finally, the predictive value of APRi for long-term outcomes, including NLS and LT, could not be adequately assessed because of substantial loss to follow-up, high mortality rate and the limited number of patients who underwent LT. External validation in larger prospective multicentre cohorts remains necessary before APRi-based prediction models can be considered for routine clinical application.

### Recommendations and future directions

Given the modest diagnostic performance observed in this study and the variability in APRi thresholds reported internationally, further prospective multicentre studies are needed for external validation of APRi across diverse populations and healthcare settings. Particular attention should be given to African cohorts and other resource-limited settings, where delayed presentation and restricted access to LT may influence clinical decision-making.

Future studies should evaluate APRi alongside other routinely available biochemical markers, imaging modalities and emerging serum biomarkers to determine its incremental value within multimodal diagnostic strategies. The development and external validation of multivariable prediction models incorporating clinical, biochemical and histological variables may further improve the non-invasive assessment of liver fibrosis in BA.

Further research is also needed to examine factors that may influence APRi values independently of liver fibrosis, including HIV exposure, antiretroviral therapy, CMV-associated BA, intercurrent hepatic inflammation and other conditions affecting serum aminotransferase levels or platelet counts. A clearer understanding of these potential confounders may improve the interpretation of APRi across different clinical settings.

Finally, future research should evaluate the relationship between APRi, long-term NLS, LT and healthcare resource utilisation to determine whether APRi-based prediction models can contribute meaningfully to clinical decision-making in BA.

## Conclusion

Aminotransferase-to-platelet ratio index should not be regarded as a replacement for established clinical decision-making in BA, including consideration of patient age, liver histology and overall clinical assessment. However, the findings suggest that APRi may provide useful adjunctive information when interpreted alongside existing clinical, biochemical and histological parameters. This study adds contextual evidence from a South African tertiary referral centre serving a resource-limited population characterised by delayed presentation, a high prevalence of CMV IgM-positive disease, HIV exposure and limited access to LT. Although the diagnostic performance of APRi was modest, the results support continued evaluation of APRi as part of multimodal non-invasive assessment strategies. Further prospective multicentre studies with external validation are required before APRi-based prediction models can be considered for routine clinical application, particularly in resource-limited healthcare settings.^35,40,41,42^

## References

[1] Hartley JL, Davenport M, Kelly DA. Biliary atresia. Lancet. 2009;374(9702):1704–1713. 10.1016/S0140-6736(09)60946-6 19914515

[2] Jimenez-Rivera C, Jolin-Dahel KS, Fortinsky KJ, Gozdyra P, Benchimol EI. International incidence and outcomes of biliary atresia. J Pediatr Gastroenterol Nutr. 2013;56(4):344–354. 10.1097/MPG.0b013e318282a913 23263590

[3] Davenport M. Biliary atresia: Outcome and management. Indian J Pediatr. 2006;73(9):825–828. 10.1007/BF02790394 17006043

[4] Haafiz AB. Liver fibrosis in biliary atresia. Expert Rev Gastroenterol Hepatol. 2010;4(3):335–343. 10.1586/egh.10.29 20528120

[5] Wildhaber BE. Biliary atresia: 50 years after the first Kasai. Int Sch Res Notices. 2012;2012:132089. 10.5402/2012/132089 PMC3523408 23304557

[6] Sinha CK, Davenport M. Biliary atresia. J Indian Assoc Pediatr Surg. 2008;13(2):49–56. 10.4103/0971-9261.43015 20011467 PMC2788439

[7] Hukkinen M, Kerola A, Lohi J, et al. Treatment policy and liver histopathology predict biliary atresia outcomes: Results after national centralization and protocol biopsies. J Am Coll Surg. 2018;226(1):46–57.e1. 10.1016/j.jamcollsurg.2017.09.009 28958913

[8] Shteyer E, Ramm GA, Xu C, White FV, Shepherd RW. Outcome after portoenterostomy in biliary atresia: Pivotal role of degree of liver fibrosis and intensity of stellate cell activation. J Pediatr Gastroenterol Nutr. 2006;42(1):93–99. 10.1097/01.mpg.0000189324.80323.a6 16385261

[9] Shneider BL, Mazariegos GV. Biliary atresia: A transplant perspective. Liver Transpl. 2007;13(11):1482–1495. 10.1002/lt.21303 17969203

[10] Serinet M-O, Wildhaber BE, Broué P, et al. Impact of age at Kasai operation on its results in late childhood and adolescence: A rational basis for biliary atresia screening. Pediatrics. 2009;123(5):1280–1286. 10.1542/peds.2008-1949 19403492

[11] Davenport M, Puricelli V, Farrant P, et al. The outcome of the older (≥ 100 days) infant with biliary atresia. J Pediatr Surg. 2004;39(4):575–581. 10.1016/j.jpedsurg.2003.12.014 15065031

[12] Brits E, Le Grange SM. Biliary atresia: The profile, management and outcome of patients treated at a tertiary hospital in central South Africa. S Afr Med J. 2023;113(11):57–62. 10.7196/SAMJ.2023.v113i11.845 38525639

[13] Davenport M, Superina R. Primary liver transplant in biliary atresia: The case for and against. J Pediatr Surg. 2024;59(8):1418–1426. 10.1016/j.jpedsurg.2024.03.005 38565475

[14] Muntean A, Kronfli R, Makin E, Davenport M. The AST-to-platelet ratio index (APRi) at Kasai portoenterostomy: Standing the test of time. J Pediatr Surg. 2023;58(12):2347–2351. 10.1016/j.jpedsurg.2023.06.012 37468346

[15] The French METAVIR Cooperative Study Group, Bedossa P. Intraobserver and interobserver variations in liver biopsy interpretation in patients with chronic hepatitis C. Hepatology [serial online]. 1994 [cited 2026 May 08];20(1 Pt 1):15–20. Available from: 10.1002/hep.1840200104 8020885

[16] Bravo AA, Sheth SG, Chopra S. Liver biopsy. N Engl J Med. 2001;344(7):495–500. 10.1056/NEJM200102153440706 11172192

[17] Kaur N, Goyal G, Garg R, Tapasvi C, Chawla S, Kaur R. Potential role of noninvasive biomarkers during liver fibrosis. World J Hepatol. 2021;13(12):1919–1935. 10.4254/wjh.v13.i12.1919 35069998 PMC8727215

[18] Ovchinsky N, Moreira RK, Lefkowitch JH, Lavine JE. Liver biopsy in modern clinical practice: A pediatric point-of-view. Adv Anat Pathol. 2012;19(4):250–262. 10.1097/PAP.0b013e31825c6a20 22692288 PMC3404724

[19] Grieve A, Makin E, Davenport M. Aspartate aminotransferase-to-platelet ratio index (APRi) in infants with biliary atresia: Prognostic value at presentation. J Pediatr Surg. 2013;48(4):789–795. 10.1016/j.jpedsurg.2012.10.010 23583135

[20] Suominen JS, Lampela H, Heikkilä P, Lohi J, Jalanko H, Pakarinen MP. APRi predicts native liver survival by reflecting portal fibrogenesis and hepatic neovascularization at the time of portoenterostomy in biliary atresia. J Pediatr Surg. 2015;50(9):1528–1531. 10.1016/j.jpedsurg.2014.11.046 25783319

[21] Yang LY, Fu J, Peng X-F, et al. Validation of aspartate aminotransferase to platelet ratio for diagnosis of liver fibrosis and prediction of postoperative prognosis in infants with biliary atresia. World J Gastroenterol. 2015;21(19):5893–5900. 10.3748/wjg.v21.i19.5893 26019453 PMC4438023

[22] Hukkinen M, Lohi J, Heikkilä P, et al. Noninvasive evaluation of liver fibrosis and portal hypertension after successful portoenterostomy for biliary atresia. Hepatol Commun. 2019;3(3):382–391. 10.1002/hep4.1306 30859150 PMC6396371

[23] Lurie Y, Webb M, Cytter-Kuint R, Shteingart S, Lederkremer GZ. Non-invasive diagnosis of liver fibrosis and cirrhosis. World J Gastroenterol. 2015;21(41):11567–11583. 10.3748/wjg.v21.i41.11567 26556987 PMC4631961

[24] Christensen E. Prognostic models including the Child–Pugh, MELD and Mayo risk scores – Where are we and where should we go? J Hepatol. 2004;41(2):344–350. 10.1016/j.jhep.2004.06.005 15288486

[25] Bourdeaux C, Tri TT, Gras J, et al. PELD score and posttransplant outcome in pediatric liver transplantation: A retrospective study of 100 recipients. Transplantation. 2005;79(9):1273–1276. 10.1097/00007890-200505150-00060 15880084

[26] Nakamura H, Tanaka E, Kaneko M, et al. The clinical importance of the trimethadione tolerance test as a method for quantitative assessment of hepatic functional reserve in patients with biliary atresia. J Clin Pharm Ther. 2001;26(6):417–424. 10.1046/j.1365-2710.2001.00370.x 11722678

[27] Wai C-T, Greenson JK, Fontana RJ, et al. A simple noninvasive index can predict both significant fibrosis and cirrhosis in patients with chronic hepatitis C. Hepatology. 2003;38(2):518–526. 10.1053/jhep.2003.50346 12883497

[28] Lind RC, Verkade HJ, Porte RJ, Hulscher JBF. Aspartate transaminase-to-platelet ratio index is not correlated with severity of fibrosis or survival in children with biliary atresia. J Pediatr Gastroenterol Nutr. 2012;54(5):698. 10.1097/MPG.0b013e318244d19d 22167019

[29] Kong F, Dong R, Chen G, et al. Progress in biomarkers related to biliary atresia. J Clin Transl Hepatol. 2024;12(3):305–315. 10.14218/JCTH.2023.00260 38426193 PMC10899875

[30] Vickers AJ, van Calster B, Steyerberg EW. A simple, step-by-step guide to interpreting decision curve analysis. Diagn Progn Res. 2019;3(1):18. 10.1186/s41512-019-0064-7 31592444 PMC6777022

[31] WeiTing C, ShuangJie L. Clinical value of transient elastography, aspartate aminotransferase-to-platelet ratio index, and fibrosis-4 in the diagnosis of liver fibrosis in children with biliary atresia. J Clin Hepatol. 2020;36(3):546–550. https://lcgdbzz.org/en/article/doi/10.3969/j.issn.1001-5256.2020.03.015

[32] Shah I, Madgum N. Correlation of APRI Index with Metavir index in children with neonatal cholestasis without biliary atresia. Ann Hepatol [serial online]. 2018 [cited n.d.];17(4):592–595. Available from: https://www.sciencedirect.com/science/article/pii/S166526811930482X 29893700 10.5604/01.3001.0012.0924

[33] Chen S, Liao B, Zhong Z, et al. Supersonic shearwave elastography in the assessment of liver fibrosis for postoperative patients with biliary atresia. Sci Rep. 2016;6(1):31057. 10.1038/srep31057 27511435 PMC4980634

[34] Kim SY, Seok JY, Han SJ, Koh H. Assessment of liver fibrosis and cirrhosis by aspartate aminotransferase-to-platelet ratio index in children with biliary atresia. J Pediatr Gastroenterol Nutr. 2010;51(2):198–202. 10.1097/MPG.0b013e3181da1d98 20531020

[35] Lampela H, Kosola S, Heikkilä P, Lohi J, Jalanko H, Pakarinen MP. Native liver histology after successful portoenterostomy in biliary atresia. J Clin Gastroenterol. 2014;48(8):721–728. 10.1097/MCG.0000000000000013 24275708

[36] McKoy JM, Bennett CL, Scheetz MH, et al. Hepatotoxicity associated with long-versus short-course HIV-prophylactic nevirapine use: A systematic review and meta-analysis from the research on adverse drug events and reports (RADAR) project. Drug Saf. 2009;32(2):147–158. 10.2165/00002018-200932020-00007 19236121 PMC2768573

[37] Delicio AM, Lajos GJ, Amaral E, Cavichiolli F, Polydoro M, Milanez H. Adverse effects in children exposed to maternal HIV and antiretroviral therapy during pregnancy in Brazil: A cohort study. Reprod Health. 2018;15(1):76. 10.1186/s12978-018-0513-8 29747664 PMC5946413

[38] Zani A, Quaglia A, Hadzić N, Zuckerman M, Davenport M. Cytomegalovirus-associated biliary atresia: An aetiological and prognostic subgroup. J Pediatr Surg. 2015;50(10):1739–1745. 10.1016/j.jpedsurg.2015.03.001 25824438

[39] Lisowska-Mikołajków D, Mikołajków A, Reczuch J, Królak-Olejnik B. [Congenital cytomegalovirus infection – still a relevant problem (based on own experience and literature)] [article in Polish]. Dev Period Med. 2018;22(1):49–57. 10.34763/devperiodmed.20182201.4957 29641421 PMC8522922

[40] Shen Q-L, Chen Y-J, Wang Z-M, et al. Assessment of liver fibrosis by Fibroscan as compared to liver biopsy in biliary atresia. World J Gastroenterol. 2015;21(22):6931–6936. 10.3748/wjg.v21.i22.6931 26078570 PMC4462734

[41] Kim S, Kang Y, Lee MJ, Kim MJ, Han SJ, Koh H. Points to be considered when applying FibroScan S probe in children with biliary atresia. J Pediatr Gastroenterol Nutr. 2014;59(5):624–628. 10.1097/mpg.0000000000000489 25003372 PMC4222704

[42] Hwang J, Yoon HM, Kim KM, et al. Assessment of native liver fibrosis using ultrasound elastography and serological fibrosis indices in children with biliary atresia after the Kasai procedure. Acta Radiol. 2021;62(8):1088–1096. 10.1177/0284185120948489 32811156

